# Mechanical properties of muscle and tendon at high strain rate in sprinters

**DOI:** 10.14814/phy2.14583

**Published:** 2020-10-10

**Authors:** Keitaro Kubo, Daisuke Miyazaki, Hideaki Yata, Naoya Tsunoda

**Affiliations:** ^1^ Department of Life Science The University of Tokyo Meguro Japan; ^2^ Faculty of Physical Education Kokushikan University Tokyo Japan; ^3^ Sports Science Laboratory Wako University Machida Japan

**Keywords:** fascicle, gastrocnemius muscle, human, ultrasonography

## Abstract

The aim of the present study was to compare the mechanical properties of muscles and tendons at high strain rates between sprinters and untrained men. Fifteen sprinters and 18 untrained men participated in this study. Active muscle stiffness of the medial gastrocnemius muscle was calculated according to changes in the estimated muscle force and fascicle length during fast stretching at five different angular velocities (100, 200, 300, 500, and 600 deg·s^−1^) after submaximal isometric contractions. Stiffness and hysteresis of tendon structures were measured during ramp and ballistic contractions. Active muscle stiffness at 500 deg·s^−1^ (*p* = .070) and 600 deg·s^−1^ (*p* = .041) was greater in sprinters than untrained men, whereas no differences in those at 100, 200, and 300 deg·s^−1^ were found between the two groups. There were no differences in stiffness or hysteresis of tendon structures measured during ramp and ballistic contractions between the two groups. These results suggest that, for sprinters, greater active muscle stiffness at a high angular velocity is caused by exercising with a high angular velocity that is typical of their training.

## Graphical abstract

More recently, we develop a specially designed motor‐driven dynamometer, in which a higher angular velocity of more than 250 deg·s^−1^ can be achieved. Using this devise, we demonstrated that active muscle stiffness at a high angular velocity was significantly greater in sprinters than untrained men.

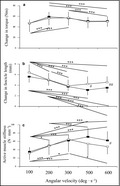

**New & Noteworthy**

More recently, we develop a specially designed motor‐driven dynamometer, in which a higher angular velocity of more than 250 deg·s^−1^ can be achieved. Using this device, we demonstrated that muscle stiffness under active condition, that is, active muscle stiffness, at high strain rate was greater in sprinters than untrained men, whereas no differences in active muscle stiffness at low angular velocity and tendon properties measured at low and high strain rates were found between the two groups.

## 1 INTRODUCTION

Over the last few decades, a considerable number of studies have been conducted to determine factors affecting 100‐m sprint performance, for example, muscle size, muscle fiber composition, and fascicle length (Costill et al., 1976; Hoshikawa et al., 2006; Kumagai et al., 2000; Lee & Piazza, 2009). Among them, several studies have used ultrasonography to investigate the mechanical properties of muscle and tendon complex in sprinters and their relation to sprint performance (Arampatzis, Karamanidis, Morey‐Klapsing, De Monte, & Stafilidis, 2007; Kubo, Ikebukuro, Yata, Tomita, & Okada, 2011; Kubo, Kanehisa, Kawakami, & Fukunaga, 2000; Miyamoto, Hirata, Inoue, & Hashimoto, 2019; Stafilidis & Arampatzis, 2007). Regarding the mechanical properties of tendons, our previous studies showed that tendon properties of knee extensors for sprinters were more compliant than those of untrained men, whereas those in plantar flexors were not (Kubo et al., 2000, 2011). Moreover, Arampatzis et al. (2007) reported that stiffness of the Achilles tendon was significantly greater in sprinters than untrained men. In these studies, however, the mechanical properties of tendons were investigated during ramp isometric contractions with a low strain rate. More recently, we observed that tendon properties (elongation and hysteresis) measured during ballistic contractions with a high strain rate were markedly different from those measured during ramp contractions (Ishigaki & Kubo, 2020; Kubo, 2018; Kubo, Ishigaki, & Ikebukuro, 2017). Furthermore, we reported that tendon elongation during ballistic contractions significantly increased after 12 weeks of plyometric training, although that during ramp contractions did not (Kubo, Ishigaki, et al., 2017). Therefore, it is likely that the mechanical properties of Achilles tendons in sprinters, who usually perform plyometric training, are different from those in untrained individuals.

We demonstrated that muscle stiffness under active conditions (i.e., active muscle stiffness) could be evaluated according to changes in joint torque and fascicle length during fast stretching (e.g., Kubo, 2014). Using this technique, we observed no difference in active muscle stiffness between sprinters and untrained men (Kubo, Miyazaki, et al., 2017). In this study, we were not able to investigate active muscle stiffness at more than 250 deg·s^−1^, since the maximal angular velocity under active conditions (i.e., during contraction) was 250 deg·s^−1^ due to the limitation of the torque motor of the dynamometer used in our previous studies (Kubo, 2014; Kubo, Ishigaki, et al., 2017; Kubo, Miyazaki, et al., 2017; Kubo et al., 2015). Moreover, it is known that the maximal angular velocity of ankle dorsiflexion during sprinting and jumping is around 500–600 deg·s^−1^ (Baba, Wada, & Ito, 2000; Struzik et al., 2015). Recent progress in technology has made it possible to improve the performance of the torque motor of the dynamometer, allowing us to develop a specially designed motor‐driven dynamometer, with which a higher angular velocity of more than 250 deg·s^−1^ can be achieved. Using this device, we may be able to detect differences in active muscle stiffness measured at a high angular velocity (around 500–600 deg·s^−1^) between sprinters and untrained men.

In the present study, we aimed to compare the mechanical properties of muscles and tendons at high strain rates between sprinters and untrained men. We hypothesized that tendon elongation and active muscle stiffness at a high strain rate would be greater in sprinters than untrained men.

## 2 METHODS

### 2.1 Subjects

The sample size was estimated using the data from our preliminary study in which the differences in active muscle stiffness between sprinters (*n* = 6) and untrained men (*n* = 6) were determined. On the basis of an α level of 0.05 and a power (1 − ß) of 0.8, it was shown that at least 15 subjects for each group were necessary for this study. The subjects for this study were 15 well‐trained male sprinters (age: 20.4 ± 0.9 years, height: 172.0 ± 4.6 cm, body mass: 67.2 ± 5.8 kg, mean ± *SD*) and 18 untrained men (age: 22.2 ± 1.8 years, height: 172.4 ± 4.6 cm, body mass: 68.2 ± 11.8 kg). All sprinters participated in sprint running competitions and were involved in regular sprint training for at least 4 years (7.2 ± 1.8). Their best official record in a 100‐m race within 1 year prior to these tests was 11.17 (*SD* 0.24) s. All untrained men were recreationally active but not engaged in any type of regular exercise program. Data on active muscle stiffness of untrained men were presented previously (Kubo et al., 2020). This study was approved by the Ethics Committee for Human Experiments, Department of Life Science (Sports Sciences), The University of Tokyo. The subjects were fully informed of the procedures to be utilized as well as the aim of the study. Written informed consent was obtained from all participants.

### 2.2 Muscle thickness and tendon cross‐sectional area

The muscle thickness of the plantar flexor muscles, that is, the medial gastrocnemius muscle (MG), lateral gastrocnemius muscle (LG), and soleus muscle (SOL), was measured at rest with an ultrasonic apparatus (SSD‐900, Aloka, Japan) as described previously (Kubo, Miyazaki, et al., 2017; Kubo et al., 2015; Kubo, Teshima, Hirose, & Tsunoda, 2014). Cross‐sectional images were obtained at proximal levels of 30% (MG and LG) and 50% (SOL) of the lower leg length. At that level, the mediolateral widths of MG and LG were determined over the skin surface, and the position of one‐half of this width was used as the measurement site for each muscle. The position of the greatest thickness in the lateral half of SOL was measured at the level described above. After the measurement of muscle thickness, the cross‐sectional area of the Achilles tendon was also measured using the ultrasonic apparatus (SSD‐6500, Aloka, Japan) at the height of the lateral malleolus (Kubo, Miyazaki, et al., 2017; Kubo et al., 2014, 2015). The repeatability of measurements of the muscle thickness and tendon cross‐sectional area was confirmed in our previous studies (e.g., Kubo et al., 2015).

### 2.3 Active muscle stiffness

A custom‐designed dynamometer (T.K.K.S‐18035, Takei Scientific Instruments Co., Ltd., Niigata, Japan) was used to evaluate active muscle stiffness at different angular velocities using a previously described procedure (Kubo et al., 2020). Subjects lay prone on a bed, with the right foot firmly secured to a footplate connected to the lever arm of the dynamometer with two straps. The ankle joint was set at 100 deg (90 deg was the neutral anatomical position where the sole of the foot was at 90 deg to the tibia, with angles of more than 90 deg on plantar flexion) with the knee joint at full extension. Subjects were asked to perform two or three maximal voluntary isometric contractions (MVC) at a 100‐deg ankle angle. The highest MVC value was used to determine the target torque during the measurement of active muscle stiffness. Maximum dorsiflexion was also performed at the same ankle angle to normalize antagonist muscle activation (see below).

After a 5‐min rest period, subjects performed the measurements of active muscle stiffness at five different angular velocities: peak angular velocities were 100, 200, 300, 500, and 600 deg·s^−1^. The dynamometer was programed to apply dorsiflexion from 100 to 80 deg. During fast stretching, subjects were instructed to maintain the activation level until the end of the movement. The order of tasks (100, 200, 300, 500, and 600 deg·s^−1^) was randomized in order to avoid any systematic effects. Periods of 140, 80, 60, 48, and 48 ms after the onset of the stretch were analyzed at 100, 200, 300, 500, and 600 deg·s^−1^ in order to equalize the analyzed range of motion among the five angular velocities (see Figure 1 of Ref. 21). In actuality, the range of motion during these periods was approximately 18.3 deg at 100, 200, and 300 deg·s^−1^ and 17.3 deg at 500 and 600 deg·s^−1^ due to the sampling rate of ultrasonic images (see below).

**FIGURE 1:**
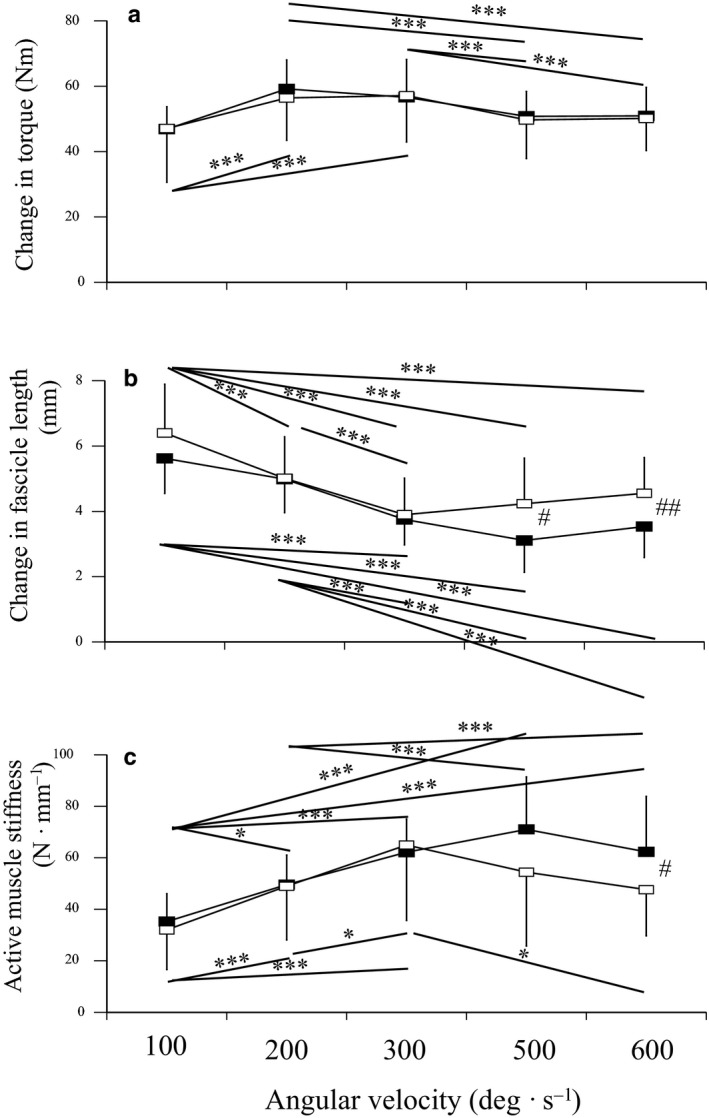
Changes in torque (A) and fascicle length (B), and active muscle stiffness (C) at 100, 200, 300, 500, and 600 deg·s^−1^ in sprinters (closed) and untrained men (open). Data are mean ± *SD*. Significant difference among the angular velocities: *p < .05, **p < .01, ***p < .001. Significant difference between sprinters and untrained men: ^#^ *p* < .05, ^##^ *p* < .01

An additional measurement for each angular velocity was performed two times at 0% MVC (relaxed condition). The averaged torque during the relaxed condition (caused by inertia and passive elasticity) was subtracted from the measured torque during the active condition (Allum & Mauritz, 1984; Blanpied & Smidt, 1992; Kubo, 2014). The measurement of active muscle stiffness was performed three times per condition (100, 200, 300, 500, and 600 deg·s^−1^) at 50% of MVC with the visual aid of exerted torque on an oscilloscope. Each test was followed by a 1–2 min recovery period. The measured values were the means of three tests. The ankle joint torque (TQ) measured by the dynamometer was converted to muscle force (Fm) by the following equation (Kubo, 2014; Kubo, Miyazaki, et al., 2017):

$$\text{Fm}=k\cdot \text{TQ}\cdot {\text{MA}}^{‐1}$$

where k represents the relative contribution of the physiological cross‐sectional area of MG within the plantar flexor muscles (Fukunaga, Roy, Shellock, Hodgson, & Edgerton, 1996) and MA is the moment arm length of the triceps surae muscles at 90 deg of the ankle joint (Maganaris, Baltzopoulos, & Sargeant, 1998).

During the measurement of active muscle stiffness, the fascicle length of MG was assessed using a real‐time ultrasonic apparatus (SSD‐6500, Aloka, Tokyo, Japan). At the level of 30% of the lower leg length, the scanning probe of the apparatus was secured with adhesive tape to the skin. Ultrasonic images were stored at 100 Hz at 100, 200, and 300 deg·s^−1^ and 125 Hz at 500 and 600 deg·s^−1^ in the computer memory of the apparatus. An electric signal was superimposed on the ultrasonic images to synchronize them with the torque, joint angle, and electromyographic activity (see below). The fascicle length was defined as the distance between the insertion of the fascicle into the superficial and deep aponeurosis. The slope of the muscle force–fascicle length in the analyzed duration was defined as active muscle stiffness (e.g., Kubo, 2014). The repeatability of the measurement of active muscle stiffness using the new dynamometer was confirmed in our previous study (Kubo et al. under submission).

Electromyographic activity (EMG) during the measurements of active muscle stiffness was recorded using a wireless EMG telemeter system (BioLog DL‐5500, S&ME, Japan) at a sampling rate of 1 kHz. Surface electrodes (DL‐510, S&ME, Japan) were placed over the bellies along the direction of fascicles of LG, SOL, and the tibialis anterior muscle (TA). The raw data were band‐pass filtered between 10 and 500 Hz. EMG was full‐wave rectified and averaged over two different phases: a 100‐ms period under five angular velocity conditions before the stretch (mEMGa) and 140‐ms period at 100 deg·s^−1^, 80‐ms period at 200 deg·s^−1^, 60‐ms period at 300 deg·s^−1^, and 48‐ms periods at 500 and 600 deg·s^−1^ after the stretch (mEMGb). The mEMGa and mEMGb values were normalized with respect to those during the measurements of MVC and are expressed as a percentage. In the present study, we failed to obtain EMG data from three subjects for sprinters and four subjects for untrained men, and mEMGa and mEMGb are presented as averages of 12 subjects for sprinters and 14 subjects for untrained men.

### 2.4 Stiffness and hysteresis of tendon structures

Stiffness and hysteresis of tendon structures at two different strain rates (see below) were measured as described previously (Kubo, 2018; Kubo, Ishigaki, et al., 2017). Subjects lay prone on the test bench of a dynamometer (custom made, VINE, Tokyo, Japan), with the right foot tightly secured to the footplate of the dynamometer by two straps. The right ankle joint was set at 90 deg with the knee joint at full extension. Prior to the test, the subject performed a standardized warm‐up and submaximal contractions to become accustomed to the test procedure. In the present study, tendon properties were measured at two different strain rates, that is, ramp and ballistic contractions. In ramp contractions, subjects were instructed to exert a gradually increasing force from a relaxed state to MVC within 5 s, followed by gradual relaxation within 5 s. In ballistic contractions, subjects were instructed to contract as strongly and rapidly as possible, followed by sudden relaxation. The tasks for ramp and ballistic contractions were repeated twice per subject with at least one minute between trials. Torque signals were analog‐to‐digital converted as a sampling rate of 1 kHz and analyzed by a computer (iMac, OS 10.14, Apple).

A real‐time ultrasonic apparatus (SSD‐6500, Aloka) was used to obtain a longitudinal ultrasonic image of MG during the contraction. Ultrasonic images were recorded on videotape at 60 Hz and synchronized with recordings of a clock timer for subsequent analyses. Displacement of the point where one fascicle was attached to the aponeurosis was considered to indicate the lengthening of tendon structures. However, tendon displacement is attributed to angular rotation and contractile tension because any angular joint rotation occurs in the direction of ankle plantar flexion during isometric contraction. To monitor ankle joint angular rotation, an electrical goniometer (Penny and Giles) was placed on the lateral aspect of the ankle. To correct the measurements taken for the elongation of tendon structures, additional measurements were performed under passive conditions. Displacement of the point at which one fascicle was attached to the aponeurosis caused by rotating the ankle from 0 to 9 deg was digitized in sonographs taken as described above. Thus, for each subject, displacement of the point where one fascicle was attached to the aponeurosis obtained from ultrasound images was corrected for that attributed to joint rotation alone (Kubo, Ishigaki, et al., 2017; Magnusson, Aagaard, Rosager, Poulsen, & Kjaer, 2001). Only values corrected for angular rotation are reported in the present study.

The torque measured by the dynamometer during isometric plantar flexion was converted to muscle force (Fm) with the same procedure as used to measure active muscle stiffness. In the present study, the muscle force and elongation of tendon structures above 50% of MVC were fitted to a linear regression equation, and the slope was adopted as tendon stiffness (e.g., Kubo, Kanehisa, Kawakami, & Fukunaga, 2000). The area within the force–elongation loop, as a percentage of the area beneath the curve during the ascending phase, was calculated as hysteresis (e.g., Kubo et al., 2011). The repeatability of measurements of the tendon structures stiffness and hysteresis measured during ramp and ballistic contractions was confirmed in our previous studies (Kubo, 2018; Kubo, Ishigaki, et al., 2017).

### 2.5 Statistics

Descriptive data included means ± *SD*. Between‐group analyses were conducted using an unpaired Student's *t*‐test. Two‐way analysis of variance (ANOVA) was used to detect the significant effects of group and angular velocity on increments in torque, changes in fascicle length, and active muscle stiffness. One‐way ANOVA was used to detect significant effects of angular velocity on mEMGa and mEMGb. If the *F* statistic of the analysis of variance was significant, differences between means were assessed using Bonferroni's post hoc test. In ANOVA, Mauchly's sphericity test was performed to assess the homogeneity of variance. Greenhouse–Geisser correction was applied where the assumption of sphericity was violated. For an unpaired Student's *t* test, the assumption of normality in the measured variables was confirmed. The effect size was calculated using partial eta‐squared (pη^2^) for one‐ and two‐way ANOVA and Cohen's *d* formula for an unpaired Student's *t* test. The level of significance was set at *p* < .05.

## 3 RESULTS

Table 1 shows the muscle thickness and tendon cross‐sectional area in the two groups. No significant differences were observed in the thickness of any measured muscles (MG *p* = .167 *d* = 0.485, LG *p* = .185 *d* = 0.478, SOL *p* = .476 *d* = 0.250) between sprinters and untrained men. In addition, no significant difference was noted in the tendon cross‐sectional area between the two groups (*p* = .957, *d* = 0.018).

**TABLE 1:** Muscle thickness and tendon cross‐sectional area in sprinters and untrained men. Mean (*SD*)

	Sprinters	Untrained men
Muscle thickness ofMG (mm)	22.4 (3.0)	20.8 (3.6)
Muscle thickness ofLG (mm)	20.5 (3.3)	18.9 (3.4)
Muscle thickness of SOL (mm)	24.3 (3.9)	23.3 (4.1)
Tendon cross‐sectional area (mm^2^)	75.0 ( 10.5)	74.8 (11.2)

Note

Abbreviations: LG, lateral gastrocnemius muscle; MG, medial gastrocnemius muscle; SOL, soleus muscle.

Regarding changes in torque during stretching, the effect of angular velocity (*p* < .001, pη^2^ = 0.366) was significant, whereas those of group (*p* = .820, pη^2^ = 0.002) and interaction between group and angular velocity (*p* = .736, pη^2^ = 0.011) were not (Figure 1a). For both groups, the increase in torque was greatest at 200 deg·s^−1^ and decreased as the angular velocity became lower or higher. Regarding changes in the fascicle length during stretching, the effect of angular velocity (*p* < .001, pη^2^ = 0.582), group (*p* = .047, pη^2^ = 0.121), and interaction between group and angular velocity (*p* = .049, pη^2^ = 0.084) were significant (Figure 1b). Although the changes in fascicle length decreased as the angular velocity became higher from 100 to 300 deg·s^−1^ for both groups, those for sprinters were significantly lower than for untrained men at 500 deg·s^−1^ (*p* = .013, *d* = 0.954) and 600 deg·s^−1^ (*p* = .008, *d* = 1.001). Regarding active muscle stiffness, the effects of angular velocity (*p* < .001, pη^2^ = 0.449) and interaction between group and angular velocity (*p* = .016, pη^2^ = 0.093) were significant, whereas that of group (*p* = .256, pη^2^ = 0.041) was not (Figure 1c). Active muscle stiffness at 500 deg·s^−1^ (*p* = .070, *d* = 0.677) and 600 deg·s^−1^ (*p* = .041, *d* = 0.743) was greater for sprinters than untrained men, whereas there were no differences in those at 100, 200, or 300 deg·s^−1^ between the two groups.

Table 2 shows the normalized mEMG values (mEMGa and mEMGb) of the three muscles during the measurement of active muscle stiffness. For all muscles, mEMGb was significantly greater than mEMGa at all angular velocities. For both groups, there were no differences in mEMGa (LG *p* = .144 pη^2^ = 0.113, SOL *p* = .105 pη^2^ = 0.199, TA *p* = .211 pη^2^ = 0.092 for sprinters; LG *p* = .258 pη^2^ = 0.095, SOL *p* = .266 pη^2^ = 0.097, TA *p* = .352 pη^2^ = 0.083 for untrained men) or mEMGb (LG *p* = .116 pη^2^ = 0.175, SOL *p* = .261 pη^2^ = 0.133, TA *p* = .118 pη^2^ = 0.182 for sprinters; LG *p* = .155 pη^2^ = 0.086, SOL *p* = .191 pη^2^ = 0.117, TA *p* = .215 pη^2^ = 0.111 for untrained men) among the five angular velocities.

**TABLE 2:** Normalized mEMGa and mEMGb of LG, SOL, and TA at 100, 200, 300, 500, and 600 deg·s^−1^ in sprinters and untrained men. Mean (*SD*)

		100 deg/s	200 deg/s	300 deg/s	500 deg/s	600 deg/s
Sprinters						
LG	mEMGa (%)	45.2 (14.1)	45.9 (14.6)	41.7 (11.0)	42.3 (13.7)	45.0 (14.6)
	mEMGb (%)	51.3 (14.9)*	52.5 (16.7)*	48.7 (18.1)*	52.6 (23.2)*	57.1 (22.9)**
SOL	mEMGa (%)	48.1 (15.8)	46.0 (14.2)	43.7 (15.7)	45.6 (11.2)	51.18 (14.6)
	mEMGb (%)	67.9 (18.3)**	71.9 (25.8)**	61.7 (22.7)*	64.3 (12.0)**	72.8 (20.9)*
TA	mEMGa (%)	4.5 (2.1)	4.9 (2.3)	4.1 (1.6)	4.0 (1.8)	5.3 (2.4)
	mEMGb (%)	5.7 (2.8)*	6.4 (3.0)**	5.5 (2.6)**	5.8 (3.1)*	7.0 (3.8)**
Untrained men						
LG	mEMGa (%)	44.0 (12.3)	45.3 (11.0)	44.7 (10.0)	43.5 (10.7)	46.9 (10.4)
	mEMGb (%)	49.3 (13.7)*	51.0 (13.8)**	53.9 (12.6)***	49.3 (12.4)**	56.0 (12.9)**
SOL	mEMGa (%)	48.2 (12.4)	49.6 (10.8)	49.6 (11.9)	46.4 (10.4)	51.1 (13.3)
	mEMGb (%)	67.2 (15.5)***	64.8 (16.2)**	69.2 (28.2)**	66.6 (21.9)**	70.3 (20.5)***
TA	mEMGa (%)	5.8 (2.7)	5.4 (2.3)	4.8 (1.7)	5.3 (2.0)	5.6 (1.8)
	mEMGb (%)	6.8 (2.0)*	6.3 (1.9)**	6.6 (2.1)***	6.4 (2.4)*	7.3 (2.3)**

Note

mEMGa and mEMGb: electromyographic activities during a 100‐ms period before the stretch and a given period after the stretch

Abbreviations: LG, lateral gastrocnemius muscle; SOL, soleus muscle; TA, tibialis anterior muscle.

* Significantly different from mEMGa (*p < .05, **p < .01, ***p < .001)

**FIGURE 2:**
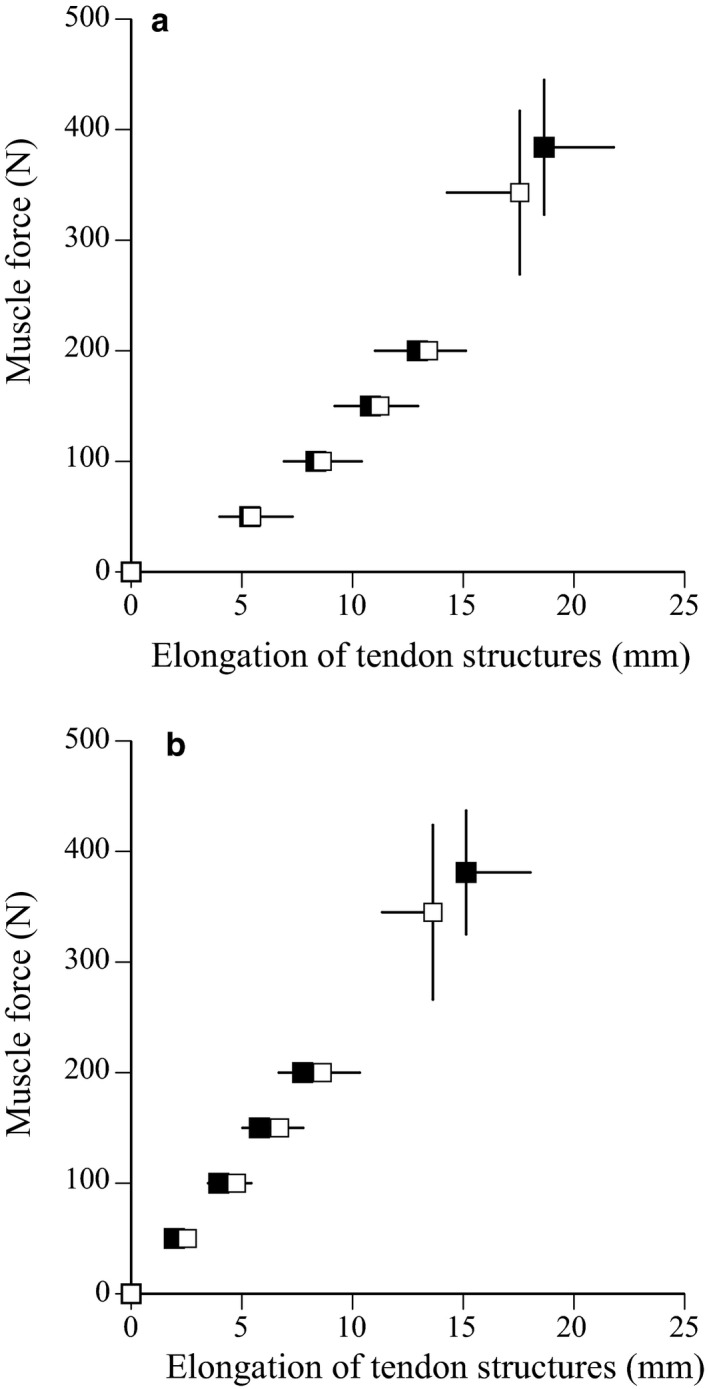
Relationship between muscle force and elongation of tendon structures during ramp (A) and ballistic (B) contractions in sprinters (closed) and untrained men (open). Data are mean ± *SD*

For both ramp and ballistic contractions, no significant differences were noted in the elongation of tendon structures between the two groups at any force production level (Figure 2). No significant differences in the maximal elongation (*p* = .447 *d* = 0.281 for ramp contraction, *p* = .113 *d* = 0.577 for ballistic contraction), stiffness (*p* = .998 *d* = 0.001 for ramp contraction, *p* = .406 *d* = 0.298 for ballistic contraction), or hysteresis (*p* = .183 *d* = 0.492 for ramp contraction, *p* = .462 *d* = 0.262 for ballistic contraction) of tendon structures were found between the two groups for ramp or ballistic contractions (Table 3).

**TABLE 3:** Mechanical properties of tendon structures in sprinters and untrained men. Mean (*SD*)

	Sprinters	Untrained men
Ramp		
Maximal elongation (mm)	18.7 (3.1)	17.9 (2.6)
Stiffness (*N*/mm)	24.8 (4.6)	24.8 (8.5)
Hysteresis (%)	15.7 (8.3)	20.6 (11.6)
Ballistic		
Maximal elongation (mm)	15.1 (2.9)	13.6 (2.3)
Stiffness (*N*/mm)	22.7 (4.8)	24.1 (4.6)
Hysteresis (%)	34.9 (13.3)	31.7 (11.1)

## 4 DISCUSSION

The present study showed that active muscle stiffness at a high angular velocity was significantly greater in sprinters than untrained men, whereas no differences in active muscle stiffness at a low angular velocity and tendon properties measured during ramp and ballistic contractions were found between the two groups. To our knowledge, this is the first study to compare muscle and tendon properties measured under a high strain rate, corresponding to that during sprinting, between sprinters and untrained men.

We previously reported that active muscle stiffness measured during slow angular velocity (peak angular velocity was 250 deg·s^−1^) for sprinters was similar to that for untrained men (Kubo, Miyazaki, et al., 2017). The present results on active muscle stiffness at 200 and 300 deg·s^−1^ agree with our previous finding. Active muscle stiffness reported by Kubo et al. (Kubo, Miyazaki, et al., 2017) represented an intrinsic muscle property without any potential neural effects, since the analyzed period (60‐ms from the onset of stretch) was selected to avoid modifying by the stretch reflex (Allum & Mauritz, 1984; Baba et al., 2000; Kubo, 2014). In this study (Kubo, Miyazaki, et al., 2017), we speculated that active muscle stiffness modified by the stretch reflex in sprinters would be greater than that in untrained men. According to the analyzed periods at 100 deg·s^−1^ (140‐ms) and 200 deg·s^−1^ (80‐ms), active muscle stiffness at 100 and 200 deg·s^−1^ in the present study would be considered to include the effect of the stretch reflex. In the present study, however, there were no differences in active muscle stiffness at 100 and 200 deg·s^−1^ between the two groups. Therefore, in contrast to our expectation in our previous study (Kubo, Miyazaki, et al., 2017), active muscle stiffness at slow angular velocities (i.e., 100–300 deg·s^−1^) in sprinters was similar to that in untrained men regardless of the effect of the stretch reflex.

An interesting finding of this study was that active muscle stiffness at 500 and 600 deg·s^−1^ in sprinters was greater than that in untrained men. Active muscle stiffness remained beyond 300 deg·s^−1^ in sprinters, whereas that decreased as the angular velocity became higher from 300 to 600 deg·s^−1^ in untrained men (Figure 1c). According to the EMG data (Table 2), we were not able to detect the effects of the short‐latency stretch reflex on active muscle stiffness at 500 and 600 deg·s^−1^ in sprinters. Moreover, previous studies demonstrated that leg and joint stiffness were greater in sprinters than long‐distance runners (Harrison, Keane, & Coglan, 2004; Hobara et al., 2008) and leg stiffness was significantly correlated with sprint performance (Chelly & Denis, 2001; Paradisis et al., 2019). Hobara et al. (2008) indicated that the greater joint stiffness in sprinters may be attributed to differences in intrinsic properties of the musculoskeletal system rather than differences in neural activities. Taking these points into account together with the present results, the previously reported greater leg and joint stiffness in sprinters might be associated with greater active muscle stiffness at a high angular velocity.

According to the previous findings (Baba et al., 2000; Struzik et al., 2015), the maximal angular velocity of ankle dorsiflexion during sprinting and jumping was around 500–600 deg·s^−1^. Moreover, we previously reported that the active muscle stiffness measured at a low angular velocity (peak angular velocity was 250 deg·s^−1^) in long‐distance runners was significantly greater than that in untrained men (Kubo et al., 2015). Several studies showed that the angular velocity of ankle dorsiflexion was around 200–300 deg·s^−1^ during endurance running (Ito, Komi, Sjodin, Bosco, & Karlsson, 1983; Tominaga, Ishii, Ueda, & Kurokawa, 2016). Considering these points, our previous findings on active muscle stiffness in long‐distance runners and sprinters (Kubo, Miyazaki, et al., 2017; Kubo et al., 2015) would be affected by exercising at different running velocities that are typical of their training. Furthermore, previous studies demonstrated the load‐ and velocity–specificity of the increase in muscular power; the plyometric training with a heavy load improved power output in the low‐velocity portion of the force–velocity curve, whereas that with a low load improved power output in the high‐velocity portion of the force–velocity curve (Kaneko, Fuchimoto, Toji, & Suei, 1983; McBride, Triplett‐McBride, Davie, & Newton, 2002; Moss, Refsnes, Abildgaard, Nicolaysen, & Jensen, 1997). This load‐ and velocity–specificity of training‐induced changes has been reported to be related to changes in muscle electrical activities (Hakkinen & Komi, 1985; McBride et al., 2002). In addition to the changes in neuromuscular activities, we may say that the load‐ and velocity–specificity for plyometric training is related to the specificity of training‐induced changes in active muscle stiffness. To clarify this point, we need to longitudinally investigate the effects of plyometric training with different kinds of load and velocity on active muscle stiffness measured at various angular velocities.

Unfortunately, we were not able to refer to the mechanisms behind greater active muscle stiffness at 500 and 600 deg·s^−1^ for sprinters in the present study. As mentioned earlier, the greater active muscle stiffness at 500 and 600 deg·s^−1^ in sprinters was not related to the excitability of the short‐latency stretch reflex. As another possible reason for the present results, we suggest that there are training‐induced changes in the mechanical properties of cross‐bridges and titin filaments within the sarcomere. However, as far as we know, no studies have investigated changes in the mechanical properties of cross‐bridges after various types of training. Regarding the changes in the mechanical properties of titin filaments, longitudinal studies showed that the titin content and isoform did not change after several weeks of plyometric training (Kyrolainen et al., 2005; McGuigan et al., 2003). Moreover, according to a cross‐sectional finding (McBride, Triplett‐McBride, Davie, Abernethy, & Newton, 2003), there was a differential expression of titin protein bands in strength and power athletes compared with untrained individuals. Regardless, further studies using isolated animal muscles are needed in order to clarify this point.

Our previous study showed that the extensibility of tendon structures during ballistic contractions significantly increased after 12 weeks of plyometric training (Kubo, Ishigaki, et al., 2017). At the beginning of the present study, therefore, tendon elongation during ballistic contractions in sprinters was expected to be greater than that in untrained men, because sprinters usually performed plyometric training. However, this hypothesis was rejected in the present study. As a reason for this difference between the hypothesis and present results, the strain rate of tendon structures during ballistic contraction (30 mm·s^−1^ on average) may be markedly lower than that during sprinting and jumping (100–200 mm·s^−1^ from Hirayama et al., 2017; Ishikawa et al., 2005). Moreover, the tendon stiffness during ballistic contractions tended to be lower than that during ramp contraction in sprinters (*p* = .075), whereas there no difference in the tendon stiffness between ballistic and ramp contractions in untrained men (*p* = .552) (statistical results not shown). Therefore, it may be that the Achilles tendon of sprinters is more compliant during sprinting and jumping compared with that measured during ramp contractions with a low strain rate.

During stretch‐shortening cycle exercises, tendon hysteresis represented the energy lost as heat due to internal damping (Butler, Grood, Noyes, & Zernicke, 1978). Hence, it would be desirable to have lower tendon hysteresis for athletes performing stretch‐shortening cycle exercises. To date, findings concerning training‐induced changes in tendon hysteresis have been conflicting. Wiesinger et al. (Wiesinger, Rieder, Kosters, Muller, & Seynnes, 2017) reported that tendon hysteresis in ski jumpers and runners was significantly lower than that in untrained individuals. Foure et al. (Foure, Nordez, & Cornu, 2010) also demonstrated that tendon hysteresis significantly decreased by 35% after 14 weeks of plyometric training. Moreover, we previously showed no change in tendon hysteresis after 12 weeks of plyometric training (Kubo, Ishigaki, et al., 2017). Considering our previous and the present results, the lack of a difference in tendon hysteresis between sprinters and untrained men and no change in tendon hysteresis after plyometric training would be related to the lower strain rate during ballistic contractions, as mentioned earlier. Unfortunately, we are currently not able to investigate tendon hysteresis during a strain rate higher than with ballistic contractions.

There were some limitations to the present study. First, we used a previously reported moment arm length in each group to calculate the muscle force. Previous studies showed that the moment arm length of the Achilles tendon in sprinters was significantly lower than that in untrained subjects (Lee & Piazza, 2009), whereas there was no difference in the moment arm length of the Achilles tendon between the two groups (Arampatzis et al., 2007). Therefore, it is possible that the calculated muscle force and tendon stiffness may be underestimated, especially for sprinters. However, the moment arm length was previously reported to change with increasing muscle force and changing joint angles (Maganaris et al., 1998). Therefore, it is difficult to obtain accurate data on the moment arm length during the measurement of muscle and tendon properties. Second, we investigated the muscle and tendon properties of only MG in the present study. Previous studies demonstrated that the mechanical properties of tendon structures in knee extensors were associated with sprint performance, although those in plantar flexors were not (Kubo et al., 2000; Stafilidis & Arampatzis, 2007). Hence, the differences in muscle and tendon properties between sprinters and untrained men may be larger in knee extensors than plantar flexors. At present, we are trying to establish a method to measure active muscle stiffness in knee extensors in our laboratory. Third, the present study involved a small sample size. Nevertheless, the results showed the differences in active muscle stiffness at a high strain rate between sprinters and untrained men. However, the best official record in a 100‐m race for sprinters was not related to any measured muscle and tendon properties in the present study (data not shown). In a future study, we need to multidirectionally investigate the effects of muscle and tendon properties on sprint performances.

In conclusion, the present study showed that active muscle stiffness at a high angular velocity (corresponding to that during sprinting and jumping) was significantly greater in sprinters than untrained men. Furthermore, there was no difference in tendon stiffness or hysteresis measured at low or high strain rates between the two groups.

## CONFLICT OF INTEREST

The authors declare that they have no conflict of interest.

## AUTHOR CONTRIBUTION

K.K, D.M, and H.Y performed the experiments. K.K, D.M, and N.T performed the data analysis. K.K and N.T wrote the initial draft of the manuscript. All authors critically reviewed the manuscript and approved the final version of the manuscript.
